# Salivary Digestion1A graduating thesis, published by recommendation of the faculty Medical Department, University of Buffalo.

**Published:** 1897-08

**Authors:** Edward J. Kiepe

**Affiliations:** Buffalo, N. Y.


### SALIVARY DIGESTION.[1]

By EDWARD J. KIEPE, Ph. G., M. D., Buffalo, N. Y.

SALIVA, the natural secretion of the mouth, is a colorless, odorless, tasteless, somewhat frothy liquid, normally of alkaline reaction ; specific gravity, 1.002 to 1.006. It serves the purpose of aiding in acts of mastication, deglutition and digestion, the latter alone concerning us here. It contains particularly, among its other ingredients, an enzyme, one of the unorganised hydrolytic ferments called ptyalin, which has the power of converting boiled starch into dextrins and maltose, which can be readily detected a moment or two after mastication.

With a view of determining exactly how quickly this conversion takes place, and also how much of a given quantity of boiled starch an equal amount of saliva would succeed in converting into maltose, the following tables of experiments were conducted.

The conditions under which these experiments were conducted are as follows : Saliva was collected and filtered to free it of débris. Boiled starch was made by mixing one part of corn starch with 200 parts of water and boiling for five or six minutes. These

---

1. A graduating thesis, published by recommendation of the faculty Medical Department, University of Buffalo.

proportions of starch and water were taken mainly for the reason that they gave a fluid product which would readily flow through burettes and pipettes.

In each experiment the saliva and boiled starch were mixed in equal proportions and allowed to stand the length of time desired at a temperature of 45° C.; then tested with Fehling's solution.

TABLE NO. 1.

One part of boiled starch + one part of saliva, tested immediately for maltose, showed .0025 per cent.

One part of boiled starch + one part of saliva, tested at the end of one minute for maltose, showed .008 per cent.

One part of boiled starch + one part of saliva, tested at the end of two minutes for maltose, showed .0175 per cent.

One part of boiled starch + one part of saliva, tested at the end of five minutes for maltose, showed .02 per cent.

One part of boiled starch + one part of saliva, tested at the end of ten minutes for maltose, showed .02 per cent.

One part of boiled starch + two parts of saliva, tested at the end of five minutes for maltose, showed .04 per cent.

The continuance of salivary digestion in the stomach is generally considered of minor importance, and such, perhaps, it may be, but nevertheless it is a question we are often called upon to decide. The facts that the four pancreatic ferments will not act unless the acid chyme from the stomach has first been rendered alkaline by the bile, and that pepsin will not act unless in an acid medium, has led many to believe that ptyalin will not act unless in an alkaline medium.

To ascertain how much maltose is formed in the stomach by the continuance of salivary digestion in the presence of an acid is no easy matter; for, to begin with, ptyalin itself is present in varying quantities, inasmuch as the percentages of maltose mentioned in Table No. 1 in each instance are averages of at least five trials. So, were we to determine the amount of maltose in the stomach after a meal of boiled starches, we would have to take into consideration the amount of ptyalin in the saliva, how much maltose had been formed in the mouth during mastication and during deglutition, and allowing that the gastric juice to have an inhibitory action on salivary digestion, how much maltose would have been formed before the gastric juice had penetrated the entire mass.

Imitating nature's way in first adding saliva to boiled starch and then adding an acid solution of the strength of normal gastric juice also presents difficulties, for the conversion of boiled starch into maltose has already begun by the time an acid solution is added, a rather short period, to be sure, but percentages of maltose obtained would vary according to how quickly one succeeded in adding the acid solution.

Adding boiled starch to acid solutions of varying strengths, and both in the same and in different proportions, boiling, allowing to stand twenty-four hours and then testing for glucose, to see if any glucose had been formed by the action of the acid, and finding none at any time, led to the method of mixing first the boiled starch and acid solution of desired strength and then adding the saliva. This method gave constant result and percentages of maltose obtained one day would nearly correspond to the same figures obtained another day, providing, of course, that the ptyalin was present in the saliva in the same amount; so, whenever a quantity of saliva had been collected and filtered for use, a portion was always taken and tested whether ptyalin was present in normal amount. By normal amount is here meant, when equal parts of saliva and boiled starch are brought together and kept at a temperature of 45° C. for five minutes and then tested for maltose, there should be present about 2 per cent. This result was reached after subjecting the saliva of different healthy people to the same test, and also that of the experimenter during a period of three months.

Boiled starch whenever made was tested to see if any glucose had been formed in the process of hydration, for glucose and maltose both react to Fehling's test, which is the test used in these experiments for the detection of maltose.

In the following tables boiled starch and saliva were prepared as before mentioned. Hydrochloric acid was used for the reason that it is the acid present in normal gastric juice in quantities of from 2 to 4 parts per 1000.

In Table No. 2 a solution of hydrochloric acid of 2 parts in 1000 parts of water was used; in No 3, 4 to 1000; in No. 4, 6 to 1000; in No. 5, 8 to 1000; in No. 6, 10 to 1000; in No. 7, 12 to 1000; in No. 8, 14 to 1000; in No. 9, 16 to 1000; in No. 10, 18 to 1000; in No. 11, 20 to 1000; in No. 12, 22 to 1000; in No. 13, 24 to 1000.

The percentages of maltose in all the tables were obtained after the mixture had stood five minutes at a temperature of 45° C.

TABLE NO. 2.

2-1000.

One part Hcl. sol. + one part boiled starch + one part saliva = .02 per cent. maltose ; two parts Hcl. sol., = .01366 per cent. maltose ; three parts, = .0082 per cent. maltose ; four parts, = .006833 per cent. maltose ; five parts, = .006307 per cent. maltose ; six parts, = .003727 per cent. maltose ; seven parts, = .003416 per cent. maltose ; eight parts, = .002921 per cent. maltose ; ten parts, = .002645 per cent. maltose ; twelve parts, = .0012615 per cent. maltose ; fourteen parts, = .001093 per cent. maltose ; sixteen parts, = none.

Comparing the percentage obtained by mixing equal parts of acid,starch and saliva with the percentage in Table No. 1, in which there are equal parts of boiled starch and saliva alone, we will notice that the percentages are the same—namely, 2 per cent. But it is when the amount of acid solution is doubled, tripled, and the like, that we notice the steady decrease in maltose.

In the following Table, No. 3, the boiled starch and saliva were obtained and used in the same manner as before; the acid solution, however, is four parts in 1000.

TABLE NO. 3.

4-1000.

One part Hcl. sol. + one part boiled starch + one part saliva, = .02 per cent. maltose ; two parts Hcl. sol., = .01366 per cent. maltose ; three parts, = .008631 per cent. maltose ; four parts, = .006833 per cent. maltose ; five parts, = .005857 per cent. maltose ; six parts, = .0041 per cent. maltose ; seven parts, = .003416 per cent. maltose ; eight parts, = .003037 per cent. maltose ; ten parts, = .0025625 per cent. maltose ; twelve parts, = .001281 per cent. maltose ; fourteen parts, = .0010933 per cent. maltose ; sixteen parts, = none.

Comparing this table with the preceding one, it will be seen that equal parts of acid sol., boiled starch and saliva yield the same percentage (2 per cent.) of maltose, and also that the decrease in amount of maltose is very nearly the same as in the preceding table, even though the acid solution is 4 in 1000.

In the following table the conditions are the same as in the preceding tables, except the acid solution, which has a strength of six parts in 1000.

TABLE NO. 4.

6-1000.

One part Hcl. sol. + one part boiled starch + one part saliva = .0164 per cent. maltose ; two parts, = .01025 per cent. maltose ; three parts, = .006833 per cent. maltose ; four parts, = .00328 per cent. maltose ; five parts, = none.

In this table the percentage of maltose is slightly lowered, being in the first experiment .0164 per cent. the rest of the percentages show the same almost regular decrease as the amount of acid is increased.

In the following table the conditions were the same excepting the acid solution, which has a strength of 8 parts in 1000.

TABLE NO. 5.

One part Hcl. sol. + one part boiled starch + one part saliva, =.0164 per cent. maltose; two parts, = .005125 per cent. maltose; three parts, = .002342 per cent. maltose; four parts, = none.

In this table, as in the preceding, equal parts of acid solution, boiled starch and saliva, give the same result, viz.: .0164 per cent. maltose. When the acid solution is doubled in amount the percentage of maltose shows a greater decrease than in the preceding table. Also in Table No. 4, five parts of acid stopped the conversion while in this Table (No. 5) four parts stop it.

In the following table, No. 6, the acid solution has a strength of ten parts in 1000.

TABLE NO. 6.

10-1000.

One part, Hcl. sol. + one part boiled starch + one part saliva, = .00455 per cent. maltose; two parts, = .00205 per cent. maltose; three parts, = none.

In the following table (No. 7) the acid solution has a strength of twelve parts in 1000.

TABLE NO. 7.

One part, Hcl. sol. + one part boiled starch + one part saliva, = .0041 per cent. maltose; two parts, = none.

In this table the decrease in percentage when equal parts are used is not so marked, while two parts of acid solution stop the conversion.

In the following table (No. 8) the acid solution has a strength of 14 parts in 1000.

TABLE NO. 8.

One part, Hcl. sol. + one part boiled starch + one part saliva, = .003565 pér cent. maltose; two parts, = none.

In the following table (No. 9) the acid solution has a strength of 16 parts in 1000.

TABLE NO. 9.

One part, Hcl. sol. + one part boiled starch + one part saliva, = .003153 per cent. maltose; two parts, = none.

In the following table (No. 10) the acid solution has a strength of eighteen parts in 1000:

TABLE NO. 10.

18-1000.

One part Hcl. sol. + one part boiled starch + one part saliva = .002645 per cent. maltose ; two parts, = none.

In the following table (No. 11) the acid solution has a strength of twenty parts in 1000 :

TABLE NO. 11.

20-1000.

One part Hcl. sol. + one part boiled starch + one part saliva = .0021578 per cent. of maltose ; two parts, = none.

In the following table (No. 12) the acid solution has a strength of twenty-two parts in 1000 :

TABLE NO. 12.

22-1000.

One part Hcl. sol. + one part boiled starch + one part saliva = .00164 per cent. maltose ; two parts, = none.

In the following table (No. 13) the acid solution has a strength of twenty-four parts in 1000 :

TABLE NO. 13.

24-1000.

One part Hcl. sol. + one part boiled starch + one part saliva = none maltose.

136 MONROE STREET.

